# Supplementary material for: Spatiotemporal Atlas of Heart Development Reveals Blood-Flow-Dependent Cellular, Structural, Metabolic, and Spatial Remodeling
Source: bioRxiv. 2025 Dec 12:2025.12.09.693024. Preprint. [Version 1] doi: 10.64898/2025.12.09.693024 (PMC12714022; doi:10.64898/2025.12.09.693024)
Supplement: 5 [file NIHPP2025.12.09.693024v1-supplement-5.pdf]

bioRxiv preprint doi: https://doi.org/10.64898/2025.12.09.693024; this version posted December 12, 2025. The copyright holder for this preprint (which was not certified by peer review) is the author/funder, who has granted bioRxiv a license to display the preprint in perpetuity. It is made available under aCC-BY 4.0 International license.

SUPPLEMENTAL FIGURES

bioRxiv preprint doi: https://doi.org/10.64898/2025.12.09.693024; this version posted December 12, 2025. The copyright holder for this preprint (which was not certified by peer review) is the author/funder, who has granted bioRxiv a license to display the preprint in perpetuity. It is made available under aCC-BY 4.0 International license.

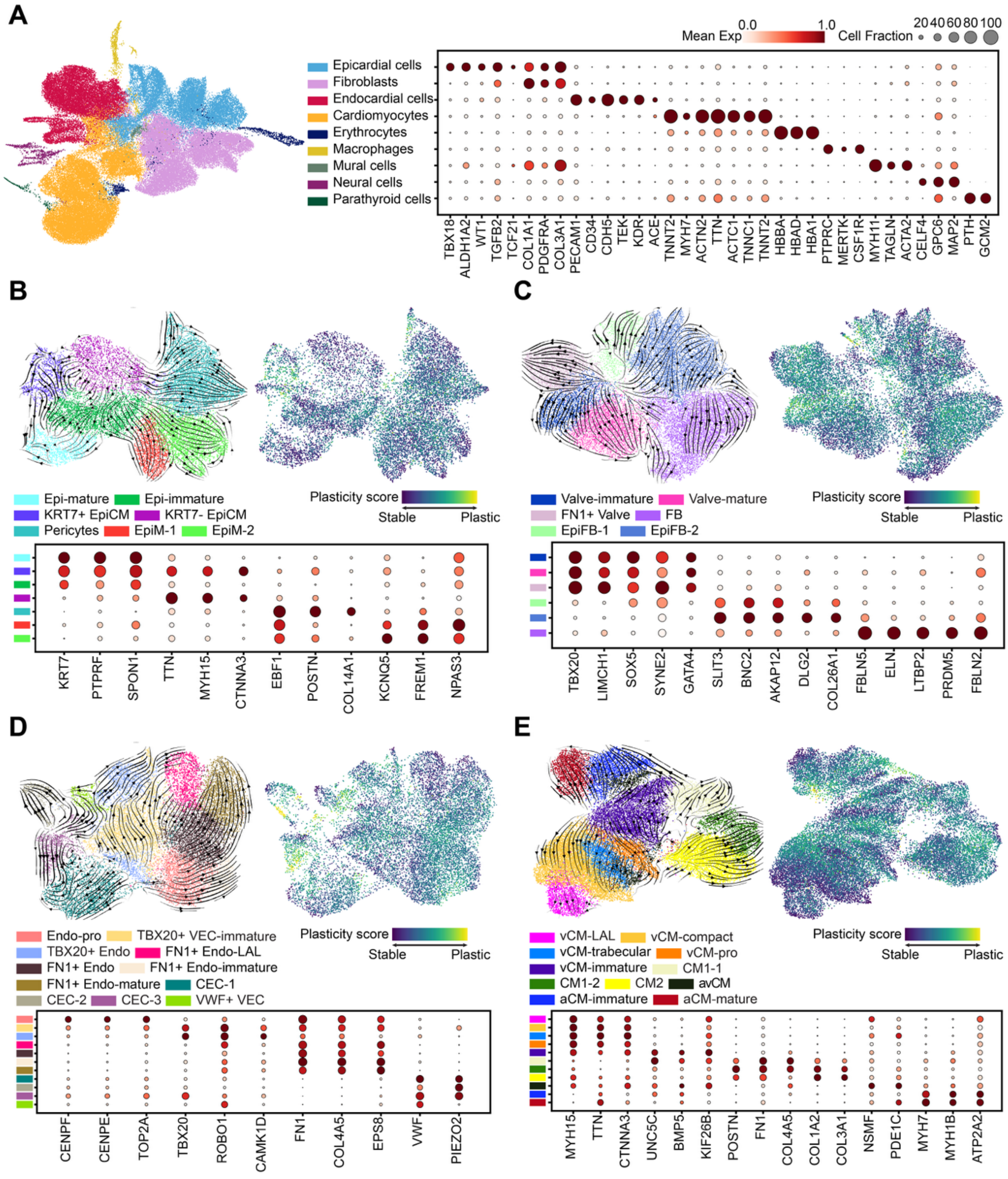

Figure S1 Cell type Annotation in scRNA-seq. (A) UMAP plot of global cell type annotation (left) and dot plot of marker genes for each cell type (right). (B) Subtype annotation of epithelial cells. UMAP plot of epithelial subtypes (left), plasticity scores overlaid with velocity embeddings (right), and dot plot of subtype marker genes. (C) Subtype annotation of fibroblasts. UMAP plot of fibroblast subtypes (left), plasticity scores with velocity embeddings (right), and dot plot of marker genes. (D) Subtype annotation of endothelial cells. UMAP plot of endothelial subtypes (left), plasticity scores with velocity embeddings (right), and dot plot of marker genes. (E) Subtype annotation of cardiomyocytes. UMAP plot of cardiomyocyte subtypes (left), plasticity scores with velocity embeddings (right), and dot plot of marker genes.

bioRxiv preprint doi: https://doi.org/10.64898/2025.12.09.693024; this version posted December 12, 2025. The copyright holder for this preprint (which was not certified by peer review) is the author/funder, who has granted bioRxiv a license to display the preprint in perpetuity. It is made available under aCC-BY 4.0 International license.

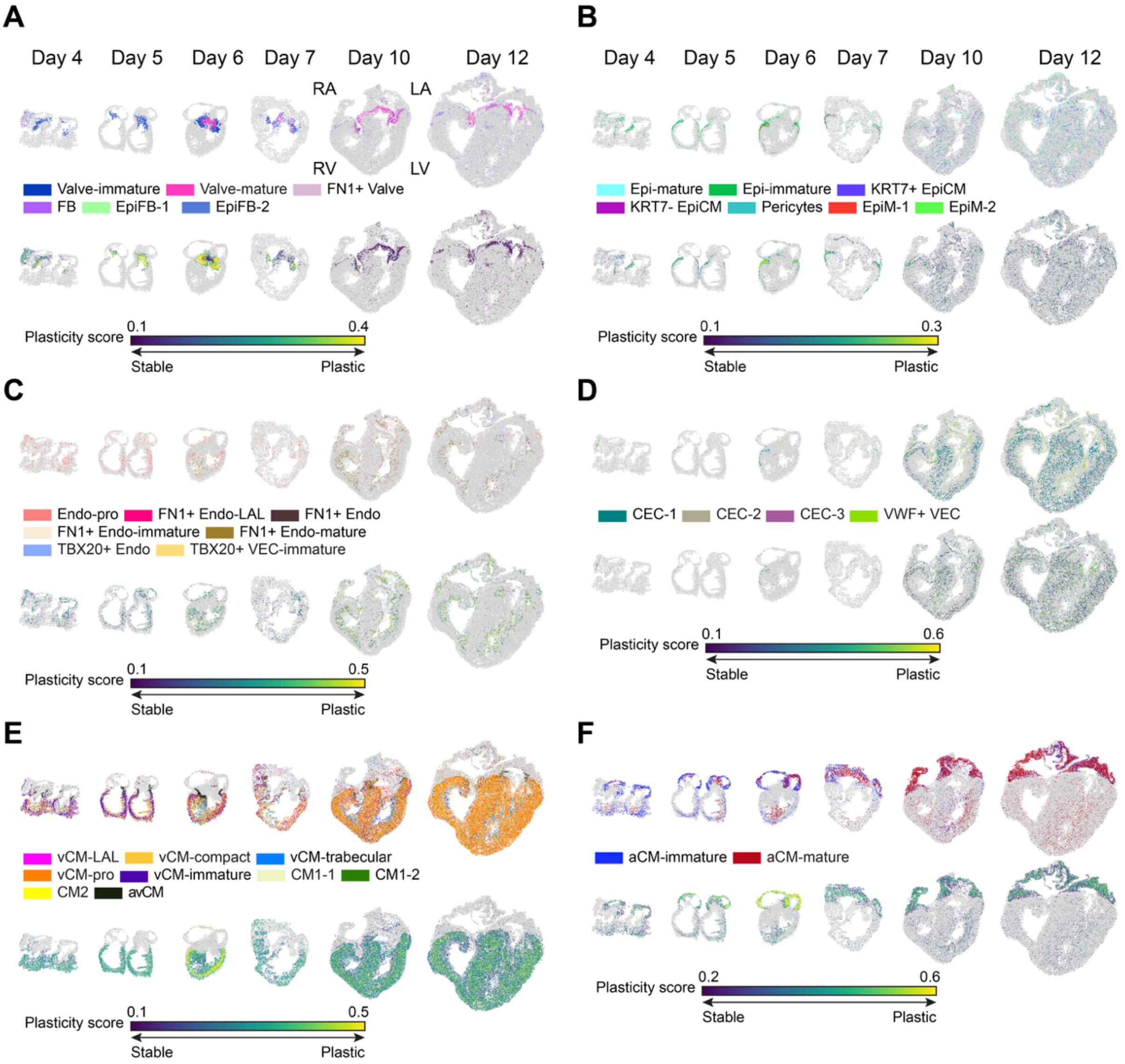

Figure S2 Spatial Cell type map and Plasticity score map in Normal heart. (A-F) Spatial plots of cell types (top) and plasticity scores (bottom) across developmental time points (Day 4, 5, 6, 7, 10, and 12) in normal hearts for (A) fibroblasts, (B) epithelial cells, (C) endocardial cells and TBX20+ VEC, (D) coronary endothelial cells (CEC) and VWF+ VEC, (E) cardiomyocytes excluding aCMs, and (F) aCMs.

bioRxiv preprint doi: https://doi.org/10.64898/2025.12.09.693024; this version posted December 12, 2025. The copyright holder for this preprint (which was not certified by peer review) is the author/funder, who has granted bioRxiv a license to display the preprint in perpetuity. It is made available under aCC-BY 4.0 International license.

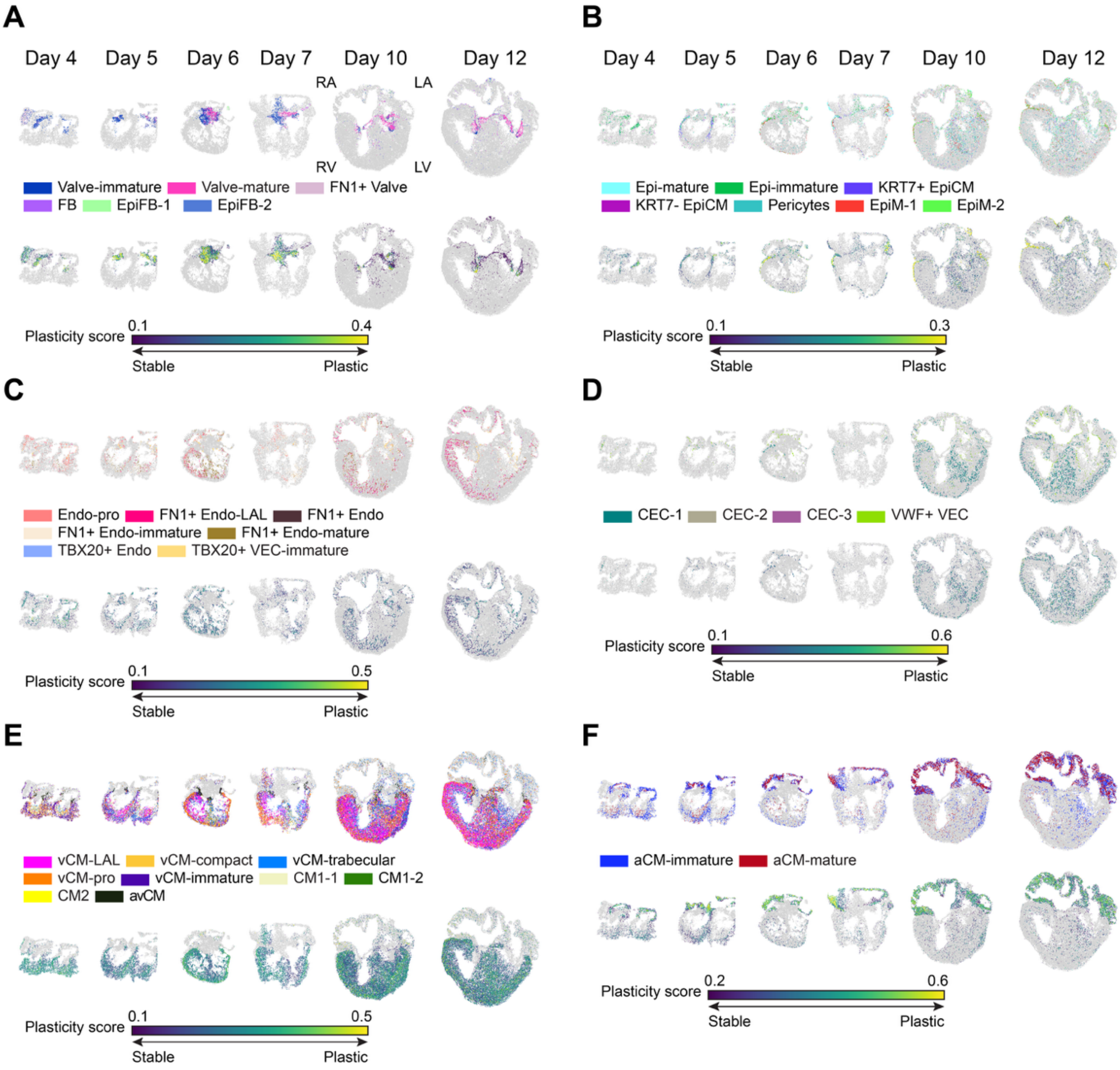

Figure S3 Spatial Cell type map and Plasticity score map in LAL heart. (A-F) Spatial plots of cell types (top) and plasticity scores (bottom) across developmental time points (Day 4, 5, 6, 7, 10, and 12) in LAL hearts for (A) fibroblasts, (B) epithelial cells, (C) endocardial cells and TBX20+ VEC, (D) coronary endothelial cells (CEC) and VWF+ VEC, (E) cardiomyocytes excluding aCMs, and (F) aCMs.

bioRxiv preprint doi: https://doi.org/10.64898/2025.12.09.693024; this version posted December 12, 2025. The copyright holder for this preprint (which was not certified by peer review) is the author/funder, who has granted bioRxiv a license to display the preprint in perpetuity. It is made available under aCC-BY 4.0 International license.

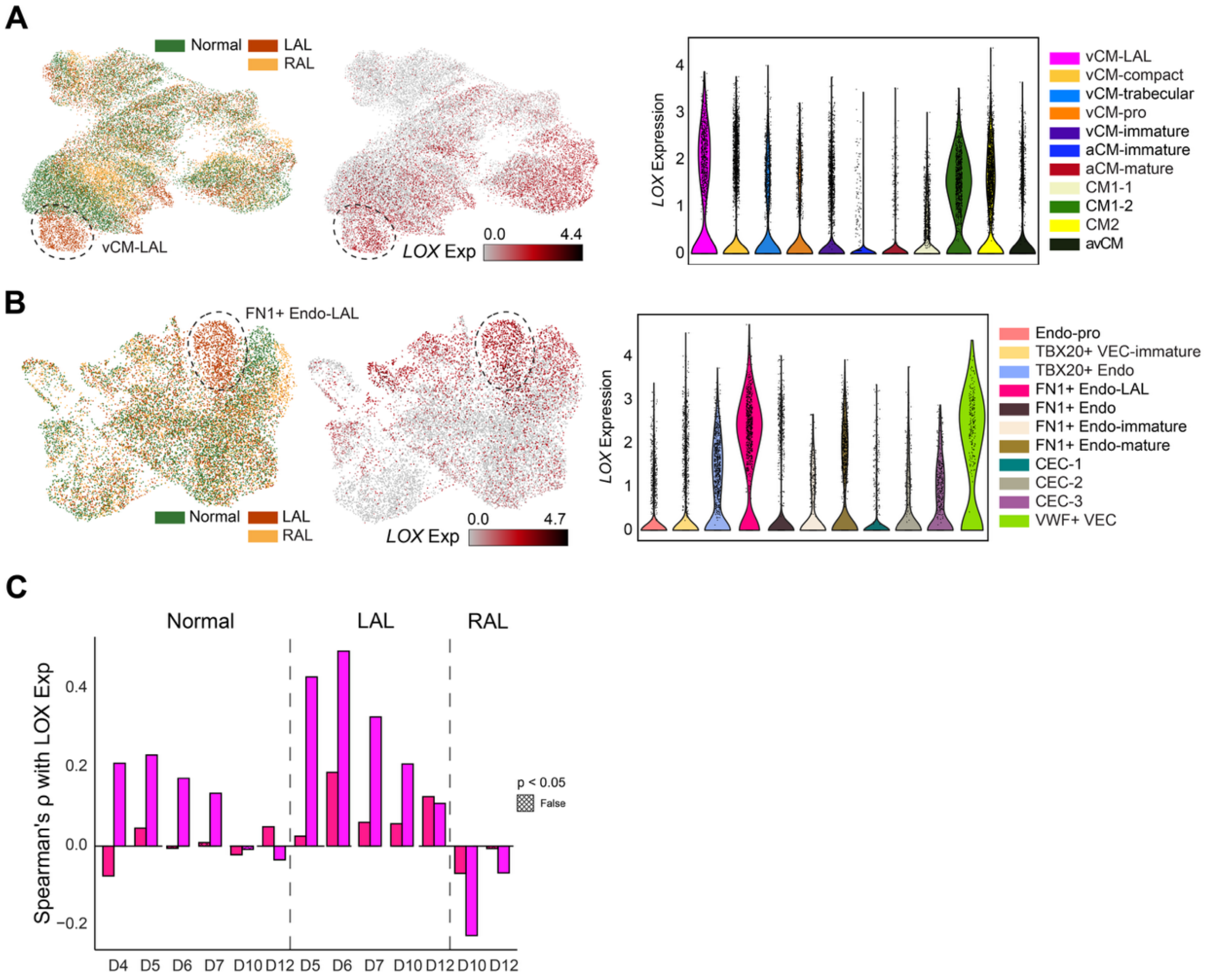

Figure S4 LOX Expressing LAL-enriched cell type. (A) UMAP plot of cardiomyocytes from scRNA-seq data, colored by condition and *LOX* expression. The vCM-LAL cluster is outlined with a dashed line (left). Violin plot of LOX expression (right). (B) UMAP plot of endothelial cells from scRNA-seq data, colored by condition and *LOX* expression. The FN1+ Endo-LAL cluster is outlined with a dashed line (left). Violin plot of LOX expression (right). (C) Bar plot of Spearman's ρ values showing correlations between *LOX* expression and the proportion of vCM-LAL or $FN1^{+}$ Endo-LAL per ST spot.

bioRxiv preprint doi: https://doi.org/10.64898/2025.12.09.693024; this version posted December 12, 2025. The copyright holder for this preprint (which was not certified by peer review) is the author/funder, who has granted bioRxiv a license to display the preprint in perpetuity. It is made available under aCC-BY 4.0 International license.

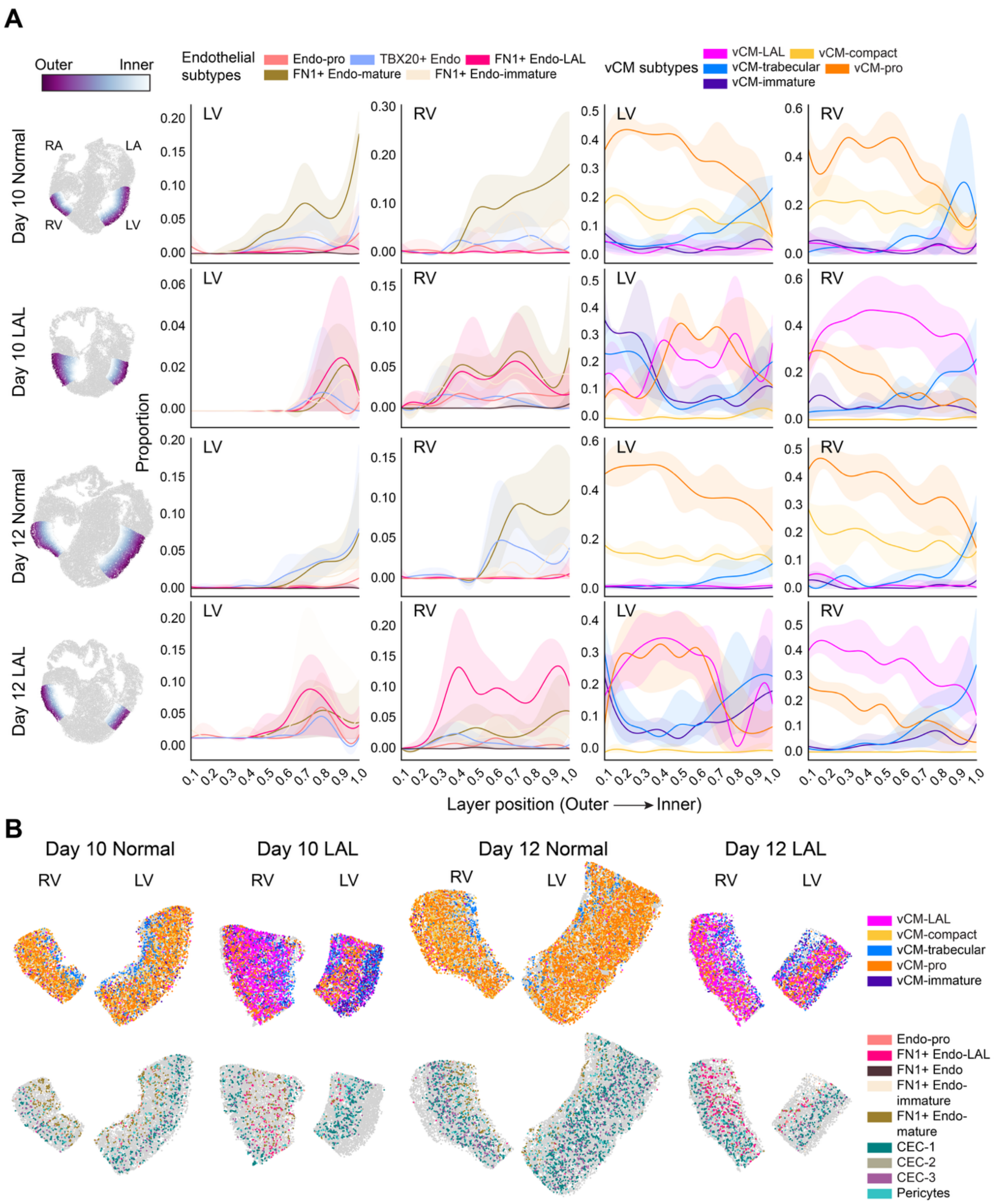

Figure S5 Identification of LOX expressing LAL-enriched cell types and Their Preferential Localization in the RV. (A) Spatial plots of Day 10 and Day 12 normal and LAL hearts colored by layer position (left). Distribution plots showing layer-wise changes in non-vascular endothelial cell and vCM proportions from outer to inner layers of the LV and RV (right). Lines represent mean proportions across five upper-bottom layer bins (**STAR Methods**), and shaded areas indicate 95% confidence intervals. (B) Zoomed in spatial maps of cell types for LV and RV on Day 10 and 12, under different conditions.

bioRxiv preprint doi: https://doi.org/10.64898/2025.12.09.693024; this version posted December 12, 2025. The copyright holder for this preprint (which was not certified by peer review) is the author/funder, who has granted bioRxiv a license to display the preprint in perpetuity. It is made available under aCC-BY 4.0 International license.

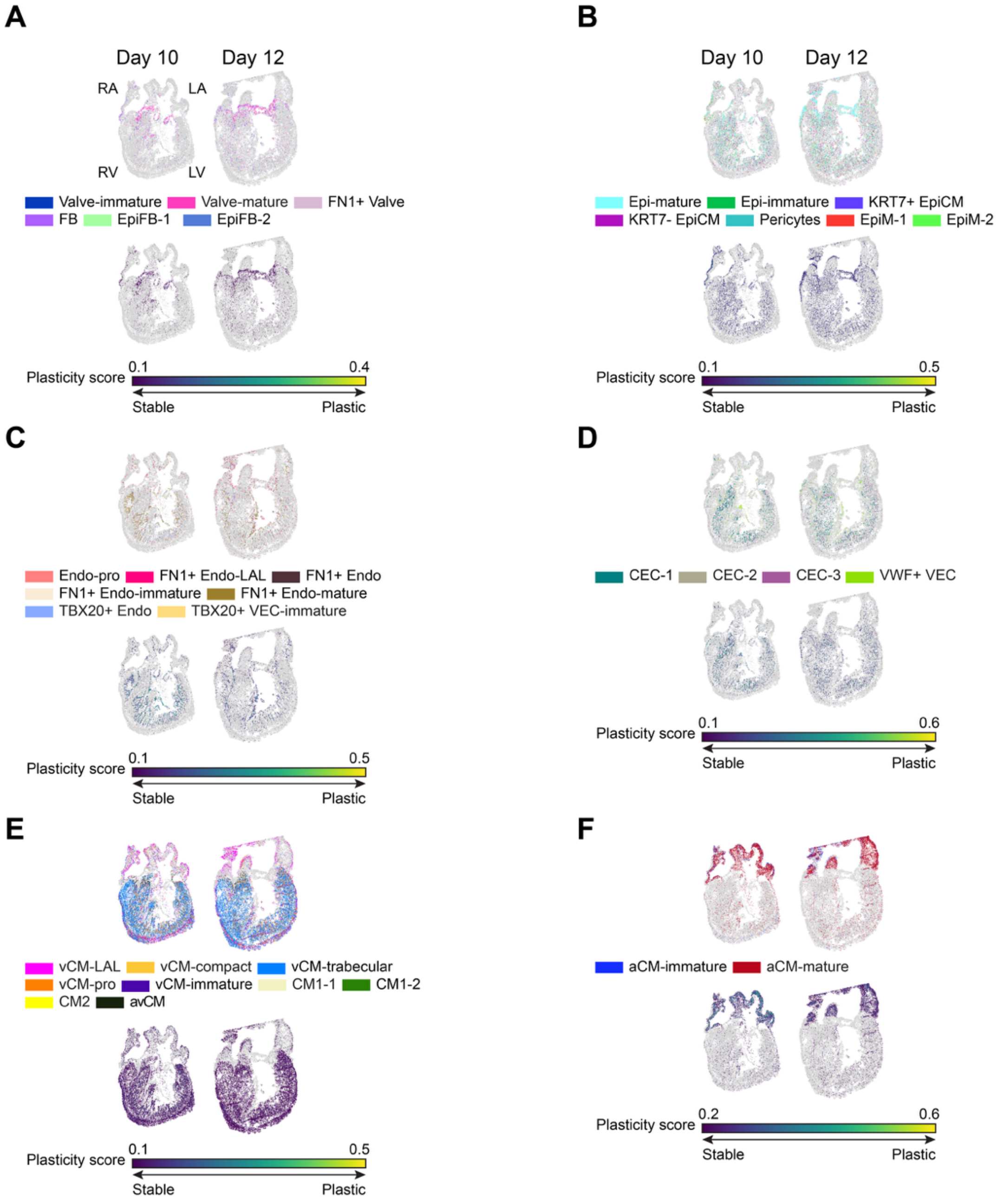

Figure S6 Spatial Cell type map and Plasticity score map in LAL heart. (A-F) Spatial maps of cell types (top) and plasticity scores (bottom) across developmental time points (Day 10 and 12) in RAL hearts for (A) fibroblasts, (B) epithelial cells, (C) endocardial cells and TBX20+ VECs, (D) coronary endothelial cells (CEC) and VWF+ VEC, (E) cardiomyocytes excluding aCMs, and (F) aCMs.

SUPPLEMENTAL INFORMATION

Table S1. Differentially expressed genes for each cell types and over-representation analysis for LAL-enriched cell types

Table S2. Differentially expressed genes for LV and RV in day 10 and 12 LAL heart

Table S3. Gene set enrichment analysis results, related Figure 5

Table S4. Differentially expressed genes comparing normal and LAL heart LV or RV in day 10 and 12, related Figure 5
